# Adolescent binge ethanol impacts H3K9me3-occupancy at synaptic genes and the regulation of oligodendrocyte development

**DOI:** 10.3389/fnmol.2024.1389100

**Published:** 2024-05-22

**Authors:** Emily R. Brocato, Rachel Easter, Alanna Morgan, Meenakshi Kakani, Grace Lee, Jennifer T. Wolstenholme

**Affiliations:** ^1^Pharmacology and Toxicology Department, Virginia Commonwealth University, Richmond, VA, United States; ^2^Alcohol Research Center, Virginia Commonwealth University, Richmond, VA, United States

**Keywords:** adolescent, binge ethanol, histone methylation, H3K9me3, myelin, oligodendrocyte maturation

## Abstract

**Introduction:**

Binge drinking in adolescence can disrupt myelination and cause brain structural changes that persist into adulthood. Alcohol consumption at a younger age increases the susceptibility of these changes. Animal models to understand ethanol’s actions on myelin and white matter show that adolescent binge ethanol can alter the developmental trajectory of oligodendrocytes, myelin structure, and myelin fiber density. Oligodendrocyte differentiation is epigenetically regulated by H3K9 trimethylation (H3K9me3). Prior studies have shown that adolescent binge ethanol dysregulates H3K9 methylation and decreases H3K9-related gene expression in the PFC.

**Methods:**

Here, we assessed ethanol-induced changes to H3K9me3 occupancy at genomic loci in the developing adolescent PFC. We further assessed ethanol-induced changes at the transcription level with qPCR time course approaches in oligodendrocyte-enriched cells to assess changes in oligodendrocyte progenitor and oligodendrocytes specifically.

**Results:**

Adolescent binge ethanol altered H3K9me3 regulation of synaptic-related genes and genes specific for glutamate and potassium channels in a sex-specific manner. In PFC tissue, we found an early change in gene expression in transcription factors associated with oligodendrocyte differentiation that may lead to the later significant decrease in myelin-related gene expression. This effect appeared stronger in males.

**Conclusion:**

Further exploration in oligodendrocyte cell enrichment time course and dose response studies could suggest lasting dysregulation of oligodendrocyte maturation at the transcriptional level. Overall, these studies suggest that binge ethanol may impede oligodendrocyte differentiation required for ongoing myelin development in the PFC by altering H3K9me3 occupancy at synaptic-related genes. We identify potential genes that may be contributing to adolescent binge ethanol-related myelin loss.

## 1 Introduction

Adolescence is a period marked by physical, behavioral, social, and cognitive changes. Cognitive changes during this time include heightened reward sensitivity, sensation seeking, impulsivity, and a diminished self-control to inhibit behaviors, all of which contribute to increased participation in risky behaviors, including the initiation and escalation of alcohol use (Spear, 2000; Casey et al., 2008; Romer et al., 2017; Lees et al., 2020). While alcohol consumption in underage youth has been on the decline over the past few decades, about 15% (5.9 million) of Americans between the ages of 12 and 20 reported current alcohol consumption in 2022 (SAMHSA, 2023). Approximately 8.3% of adolescents ages 12 to 20 (3.2 million teens) did so in the form of binge drinking in the past month, defined as having five or more drinks on one occasion (SAMHSA, 2023). Binge drinking during adolescence can be particularly harmful, as the brain is still undergoing processes such as myelination and synaptic pruning (Spear, 2000). Alcohol use can delay or disrupt this ongoing development, resulting in many structural, cognitive, and behavioral effects (De Bellis et al., 2005; Medina et al., 2008; Bava et al., 2013; Squeglia et al., 2015; Lees et al., 2020). Adolescents respond differently to alcohol than their adult counterparts, showing increased sensitivity to the rewarding aspects of alcohol, and decreased sensitivity to the aversive aspects of alcohol (Spear, 2014). This enables adolescents to increase their alcohol consumption, giving rise to the binge drinking behavior commonly seen among this age group, while subsequently increasing the risk for developing an alcohol use disorder (AUD) later in life (Grant and Dawson, 1997; Spear, 2000).

Human brain imaging studies have shown that consuming alcohol in adolescence can disrupt myelination and cause brain structural changes, finding reductions in prefrontal cortex (PFC) (De Bellis et al., 2005; Medina et al., 2008), white matter (Luciana et al., 2013; Pfefferbaum et al., 2018; El Marroun et al., 2021) and myelin fiber tract volumes and integrity (McQueeny et al., 2009; Bava et al., 2013; Elofson et al., 2013). Binge drinking in teens is associated with thinning of cortical and subcortical regions that play a key role in memory, attention, language, awareness and consciousness (Cservenka and Brumback, 2017). Likewise, teens with an alcohol misuse disorder encounter cognitive deficits in visuospatial functioning or reading recognition, some of which persist after abstinence (Brown and Tapert, 2004). This effect may be dose-dependent, as diminished PFC white matter was negatively correlated with alcohol consumption (De Bellis et al., 2005). The age of onset for binge drinking also affects the susceptibility to these white matter changes. In a study of adult veterans, those who reported binge drinking at an early age (<15 years old, i.e., early onset binge drinkers) had decreased white matter integrity in frontostriatal circuits that mediate inhibitory control compared to late onset binge drinkers (Knoff et al., 2023).

Similar outcomes have been characterized in rodent studies, as adolescent binge ethanol has induced frontal neurodegeneration (Crews et al., 2000), loss of neurogenesis (Crews et al., 2006), decreased myelin expression (Montesinos et al., 2015; Wolstenholme et al., 2017), altered myelin structure (Tavares et al., 2019), and alterations in oligodendrocyte (OLG) differentiation (Guo et al., 2021). Myelin fiber density is decreased (Vargas et al., 2014) and the intralaminar sheath is damaged in young rodents exposed to intermittent ethanol and persists into adulthood (Montesinos et al., 2015). Lowered myelin expression and formation disrupts axonal integrity and can put individuals at risk for continued AUDs (Bava et al., 2009; Harper, 2009). In adult animals, chronic ethanol delays OLG maturation (Chiappelli et al., 1991), reduces the proliferation, differentiation, and survival of premyelinating OLGs, and decreases myelin basic protein (MBP) expression in the mPFC (Richardson et al., 2009; Kim et al., 2015). Some of these changes reverse upon protracted ethanol abstinence (Navarro and Mandyam, 2015). Similarly, in adolescent rodents, mature OLGs and grey matter myelination in the infralimbic and prelimbic PFC decreased shortly after binge ethanol (from postnatal day (PND) 26–52), but increased in ethanol groups in adulthood during abstincence (Liu et al., 2021). In a shorter adolescent binge paradigm (PND 28–37), grey matter myelin in the medial PFC (mPFC) and hippocampus was reduced after ethanol binges, but this did not persist in adult mPFC (Rice et al., 2019). Adult ethanol decreased the number of newly generated mature OLGs and there was reduced expression of HDAC1 in new oligodendrocyte precursor cells (OPCs), suggesting that ethanol alters the epigenetic programming on OLG maturation (Guo et al., 2021). While it is known that alcohol can alter the developmental trajectory of the brain in regards to myelination and structure, the precise trajectory of these changes and the molecular factors underlying their development, however, remain elusive.

Epigenetic regulation of gene expression provides an attractive mechanism for driving changes in myelin structure after binge ethanol. Epigenetic modifications can positively or negatively impact gene transcription, and are dynamic marks that are affected by environmental stimuli, such as stress or drugs of abuse (Jaenisch and Bird, 2003; Liu et al., 2008; Karlić et al., 2010; Hitchcock and Lattal, 2014). There is a growing body of literature that demonstrates epigenetic dysregulation in the brain after alcohol exposure in both humans (Ponomarev et al., 2012) and rodent models (Barnea-Goraly et al., 2005; Pascual et al., 2009, 2012; Warnault et al., 2013; Barbier et al., 2015; Pandey et al., 2015; Montesinos et al., 2016; Wolstenholme et al., 2017; Kyzar et al., 2019; Bohnsack et al., 2022; Brocato and Wolstenholme, 2023) (as reviewed in Brocato and Wolstenholme, 2021), and this dysregulation provides a pathway that could result in the loss of myelin.

OLGs are the main myelinating cell in the brain, and the differentiation of OLGs into their mature, myelinating form is under epigenetic control. OPCs arise from multipotent neuroepithelial progenitor cells in the developing central nervous system (CNS) (Emery and Lu, 2015). OPCs are mobile and highly proliferative, and once they arrive in their final position, they can become OLGs via terminal differentiation (Emery and Lu, 2015). DNA methylation (Jackson et al., 2004), histone acetylation (Cunliffe and Casaccia-Bonnefil, 2006; Ye et al., 2009; Dai et al., 2015b; Zhang et al., 2018) and histone methylation (Sher et al., 2008; He et al., 2017) have all been shown to play a role in OLG differentiation processes, and epigenetic disruption of these processes underlies myelin loss in pathologic states such as multiple sclerosis (Pedre et al., 2011; Huynh et al., 2014) and Rett Syndrome (Nguyen et al., 2013; Sharma et al., 2015). OPCs are electrically responsive cells, and the differentiation of OPCs into mature, myelinating OLGs is characterized by progressive repression of genes associated with electrical responsiveness and activation of genes responsible for producing structural components of myelin (Liu et al., 2015). Tri-methylation of histone 3, lysine 9 (H3K9me3) was found to be essential for this process (Liu et al., 2015), and an increase in H3K9me3 is correlated with an increase in myelin (Chen et al., 2020). Increased H3K9me3 at specific loci is responsible for repression of genes related to membrane excitability, and was found to reduce the electrical activity of OPCs to allow for their differentiation into functional OLGs (Liu et al., 2015).

Alterations in H3K9me2 have been associated with drugs of abuse, such as cocaine (Maze et al., 2010), and H3K9me3 dysregulation was found to be a result of adolescent binge ethanol (Wolstenholme et al., 2017). H3K9me1 and *Prdm2*, the enzyme responsible for H3K9 mono-methylation, were decreased in the PFC of adult dependent rats (Barbier et al., 2016). Reduction of *Prdm2* in the PFC of non-dependent rats increased ethanol drinking even in the presence of quinine adulteration, displaying phenotypes characteristic of alcohol dependence (Barbier et al., 2016). The histone demethylase, *Lsd1*, was altered by adolescent ethanol exposure and H3K9me2 protein was increased in the amygdala of adults following adolescent ethanol (Kyzar et al., 2016). Previously, our lab used an unbiased transcriptomics approach to investigate the possible mechanisms of reduced myelin associated with adolescent binge ethanol (Wolstenholme et al., 2017). Altered levels of histone methyltransferases specific for the H3K9me3 mark, as well as decreased expression of myelin-related genes were found in the PFC of adolescent mice treated with binge ethanol (Wolstenholme et al., 2017). Together, this data suggests that adolescent binge ethanol negatively impacts oligodendrocytes, and that this deficit may be epigenetically regulated through an oligodendrocyte differentiation pathway. To further investigate ethanol-induced epigenetic changes and learn how they influence myelin, we used a series of chromatin immunoprecipitation and qPCR assays to assess H3K9me3 and gene expression changes in the PFC after adolescent binge ethanol. We conducted chromatin immunoprecipitation coupled to sequencing (ChIP-seq) for H3K9me3 to identify global changes to H3K9me3 in the PFC after adolescent binge ethanol treatment. We also conducted qPCR time courses to measure myelin-related gene expression changes in the PFC. To focus our attention on oligodendrocyte differentiation-specific effects, we used O4+ magnetic cell separation to analyze OPC and OLG lineage cells within the PFC. O4+ cells were assayed in a combined time course and dose response to assess expression changes in genes involved in the transcriptional and epigenetic regulation of myelin and the structural components of myelin. These assays helped to unveil potential genes that may be contributing to adolescent binge ethanol-related myelin loss.

## 2 Materials and methods

### 2.1 Animals

In all experiments, males and female adolescent DBA/2 J mice were used. DBA/2 J mice were used as they show a more robust acute response to binge ethanol than the commonly used C57BL/6 J strain—DBA/2 J mice show increased sensitivity to ethanol in a loss of righting reflex test, as well as increased locomotor activity compared to C57/B6 mice (Phillips et al., 1994; Kerns et al., 2005). Importantly, this strain also shows a more prominent decrease in myelin gene expression (Kerns et al., 2005; Goudriaan et al., 2020)—important to our model as myelin decreases have been widely shown in human studies of adolescent binge drinking (De Bellis et al., 2005; Medina et al., 2008; Pfefferbaum et al., 2018; El Marroun et al., 2021). Male and female DBA/2 J mice arrived from Jackson Laboratories to the Virginia Commonwealth University vivarium at postnatal day 19–21 (Bar Harbor, ME, United States) for Experiments 1 and 2. Adult male and female DBA/2 J breeding trios (n = 6 males and 12 females) were obtained from Jackson Laboratories for the O4+ time course and dose response in Experiment 3. Mice were housed 4/cage in same sex cages in an AALAC-accredited facility under 12 h light/dark cycles with food and water available *ad libitum* for the entire experiment. After a week to acclimate to the animal facility, mice were habituated to the oral gavage procedure with 0.1% saccharin on PND 27 and 28, then divided into two treatment groups: ethanol treated and control. Males and females were orally dosed via gavage with 4 g/kg ethanol (25% w/v in water) or water intermittently on PND 29, 30, 33, 34, 37, 38, 41, and 42. PFC tissue was collected 24 h after the last dose of ethanol (3–6 h into the animals’ light cycle), based on a previous study in our lab where we saw significant decreases in H3K9 and myelin-related gene expression at this timepoint (Wolstenholme et al., 2017). A triangle-shaped wedge of tissue above the corpus callosum was collected at Bregma 0.5 mm to represent the PFC, consistent with typical PFC dissection regions for the Wolstenholme lab (Wolstenholme et al., 2017; Brocato and Wolstenholme, 2023). This wedge contains prelimbic, infralimbic and medial PFC as well as anterior cingulate. For the time course in Experiment 3, mice were harvested 24 h after one, four, or eight ethanol doses, or 3 weeks after the full eight doses—thus, not all mice in this study were subjected to the full eight doses of ethanol or water. Frontal pole was collected and O4+ cells were enriched from the input cells using an antibody-based magnetic bead system. All animal housing and care was conducted with the approval of the Virginia Commonwealth University IACUC Committee and in accordance with the NIH Guide for the Care and Use of Laboratory Animals.

**Figure 1:**
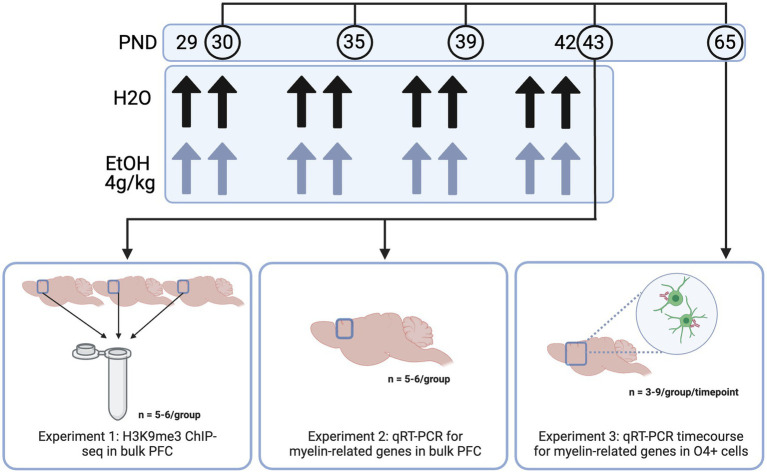
Methods. Adolescent DBA/2 J mice were dosed with water or ethanol (4 g/kg) from PND 29–42. PFC tissue was dissected (Experiments 1 and 2) or frontal pole tissue was collected and subjected to O4+ separation (Experiment 3) for downstream analyses. Created with BioRender.com.

In Experiment 1, we aimed to determine the genes at which adolescent binge ethanol dysregulated H3K9me3, a histone mark required for oligodendrocyte differentiation, using ChIP-seq (Liu et al., 2015). Here, male and female mice were dosed with ethanol or water from PND 29–42 as described above and PFC tissue was harvested 24 h after the last dose of ethanol. For this study, 66 total animals were used. To ensure enough DNA input for high quality sequencing data, we combined three PFCs from each treatment group and sex into a single sample. Each combined tissue sample was homogenized and divided in half between ChIP-seq and RNA-seq sample preparations (RNA-seq data disseminated in Brocato and Wolstenholme, 2023 and at GEO GSE220745), so that we were able to link chromatin-level changes with gene expression-level changes from within the same tissue. Five biological replicates (of three pooled PFCs) were obtained for water-treated male and female groups, and six biological replicates (of three pooled PFCs) were obtained for ethanol-treated males and females, resulting in *n* = 22 pooled samples for Experiment 1 (Figure 1).

In Experiment 2, we aimed to determine expression of genes involved in the transcriptional regulation of oligodendrocyte development and the structural components of myelin immediately (4 h) and 24 h after the last dose of ethanol using qPCR. Male and female mice (n = 5−6/group for each timepoint) were dosed from PND 29–42 and PFC tissue was collected 4 and 24 h after the last ethanol dose.

In Experiment 3, we aimed to determine whether adolescent binge ethanol altered oligodendrocyte differentiation. Here, we dosed male and female mice (*n* = 3−9/group per timepoint) as described above, and collected frontal pole tissue 24 h after one, four, or eight doses of water or ethanol (PND 30, 35, or 43) and three weeks after the last dose of ethanol (PND 65). Frontal pole tissue (collected as cortical tissue rostral to Bregma 0.5 mm, excluding the olfactory bulbs) was then subjected to O4+ separation and qPCR to assess gene expression changes in myelin-related genes over the course of our dosing paradigm. One mouse from each litter per sex was used at each timepoint and treatment group to reduce potential litter effects.

### 2.2 Chromatin immunoprecipitation

Prior to ChIP, the H3K9me3 antibody (Abcam #ab8898) was validated using qPCR to verify H3K9me3 presence or absence at specific genomic locations (Supplementary Figure S1). The same antibody lot number was used for all ChIP reactions. H3K9me3-ChIP was performed on PFC tissue using the MAGNA ChIP Hi-Sens kit (Millipore), following the manufacturer’s instructions. Briefly, tissue from 3 combined PFCs was dounce homogenized on ice, divided in half, and pelleted. One half of each sample was flash frozen and reserved for RNA-seq (Brocato and Wolstenholme, 2023). The other half of each sample was subjected to fixation, where DNA was cross-linked to histones by incubating cells in a 1% formaldehyde solution at 37°C for 10 min. The formaldehyde reaction was quenched using glycine, and tissue was washed 3x with PBS containing a protease inhibitor. Cell lysis buffer was added to isolate nuclei. Prepared chromatin was sonicated using a Bioruptor Pico (Diagenode) for 12 min (30 s on/30 s off). Fragment size was verified on 2% agarose gel to be between 200–1,000 bp. After sonication, samples were stored at −20°C to await immunoprecipitation, and a portion of sonicated chromatin from each sample was reserved as an input reference. For immunoprecipitation, sonicated chromatin was incubated overnight with 1 μg of H3K9me3 antibody bound to Protein A/G magnetic beads with end-over-end rotation. After incubation, DNA was eluted from beads in elution buffer with proteinase K. DNA was extracted using phenol:chloroform:isoamyl alcohol (ThermoFisher) and purified with ethanol precipitation. Pellets were resuspended in 20 μL TE buffer and stored at −20°C. To obtain sufficient DNA for sequencing, 8 technical replicates from each sonicated sample were subjected to immunoprecipitation. DNA from all 8 technical replicates were combined and concentrated using a vacuum prior to library preparation. Independent control samples were run alongside each set of ChIP reactions to ensure successful chromatin immunoprecipitation.

### 2.3 ChIP-seq library preparation and sequencing

ChIP-seq data have been deposited with the Gene Expression Omnibus resource (Accession #GSE220746). To avoid non-biological experimental variation that could arise from sample batches, samples were randomized prior to each processing stage (sonication, ChIP, library preparation, and lane assignment). Input and H3K9me3-enriched samples were sequenced by the VCU Genomics Core facility for library preparation and sequencing. Preparation of libraries were conducted following standard protocols using the Accel-NGS 2S PLUS DNA Library kit (Swift). Samples were sequenced on a NextSeq 2000 (Illumina) using 150 base pair single-end reads at greater than 50 million reads. A summary of ChIP-seq metrics can be found in Supplementary Table S1.

### 2.4 ChIP-seq alignment, differential binding analysis, and bioinformatics analysis

Quality of FASTQ formatted samples files were assessed with FastQC. Adapter sequences and reads with quality scores <20 were removed using CutAdapt (Martin, 2011) and aligned using Bowtie2v2.4.1 (Langmead and Salzberg, 2012) with GRCm38/mm10 reference genome. Reads that aligned to blacklisted regions were discarded, and only reads that mapped to a single location were used in subsequent analysis. Peaks were identified using SICERv1.1 (W200, G400). Sex-specific responses to ethanol have been well-noted (Squeglia et al., 2011; Labonté et al., 2017; Wolstenholme et al., 2017; Finn et al., 2018; Tavares et al., 2019; Flores-Bonilla and Richardson, 2020; Hitzemann et al., 2020; Radke et al., 2021; Bent et al., 2022), and because of this, we had an *a priori* hypothesis that ethanol induces different molecular changes in males and females. Thus, our male and female ChIP-seq analyses were run separately. These sex-specific responses are exemplified with previous data from our lab showing males had more robust decreases in myelin-related gene expression (Wolstenholme et al., 2017). These sex-specific responses have been seen specifically in other sequencing studies, where males and females show little overlap in differentially expressed genes (Labonté et al., 2017; Finn et al., 2018). Another recent ethanol-associated RNA-seq study also chose to analyze the sexes separately, given the sex-specific effects in alcohol use (Hitzemann et al., 2020). Normalization and differential peak binding analyses were conducted using the Bioconductor R package DiffBind v3.4.11. DiffBind identified sites that were differentially bound between sample groups. DiffBind’s statistical analysis routine relies on the Bioconductor package DESeq2, which utilizes a negative binomial generalized linear model. This analysis assigned a *p*-value and false discovery rate (FDR) to each candidate binding site indicating confidence that they were differentially bound. Genes with a *p*-value ≤0.05 were considered significantly altered and used in downstream bioinformatic analysis to expand our gene list for hypothesis-generating analyses.

Gene and genomic region annotations were obtained using the Bioconductor package ChIPSeekerv1.28.3. Representative samples of H3K9me3 read coverage are shown in Supplementary Figure S1. Genomic region annotations of the peaks differentially bound by H3K9me3 due to ethanol are shown in Supplementary Figure S2.

**Figure 2:**
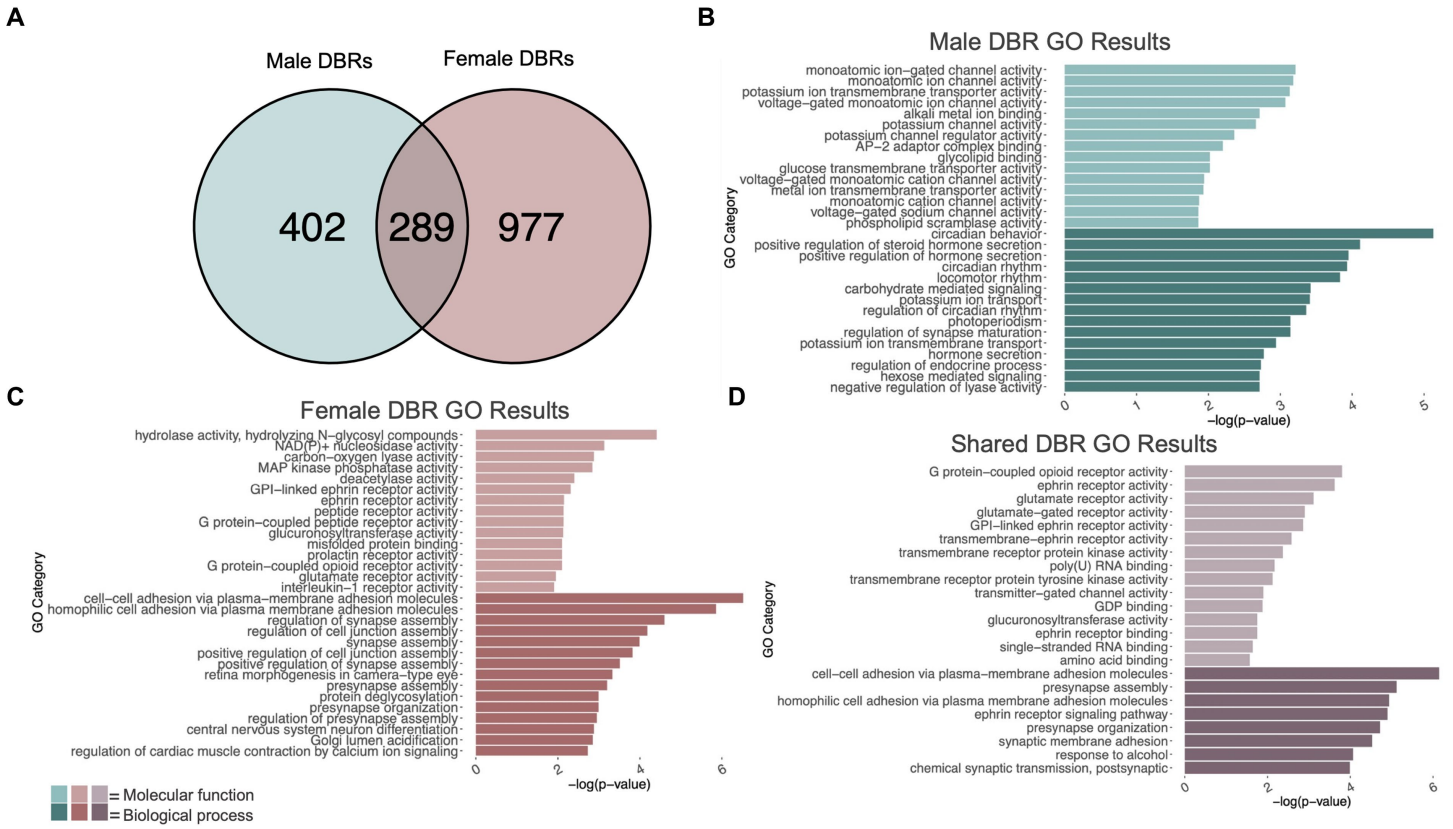
GO analysis of genes showing differentially bound H3K9me3 regions (DBR). **(A)** number of genes showing differential H3K9me3 due to adolescent binge ethanol in males and females at *p* < 0.05. Fisher’s exact test determined male and female gene overlap to be significant, *p* = 2.9e-131. **(B)** GO analysis of genes showing differential trimethylation of H3K9 unique to males. **(C)** GO analysis of genes showing differential trimethylation of H3K9 unique to females. **(D)** GO analysis of genes showing differential trimethylation of H3K9 that were shared between males and females.

Gene ontology over-representation analysis was determined using ToppFun (Chen et al., 2009). Background gene sets were used to prevent enrichment based on tissue. Detected genes for males (Supplementary Table S3, column L) and females (Supplementary Table S3, column Y) were used as background for the male and female gene ontology analyses, respectively. For the shared gene ontology analysis, a shared detected gene set was used for the background (i.e., the intersection of genes contained in Columns L and Y, Supplementary Table S3). Gene sets were filtered based on the number of genes within each category (min = 4, max = 500) and *p*-value of ≤0.05. Lists were further filtered by gene list hits – categories containing <3 hits were removed. Categories that had identical query gene lists and similar category names were removed to reduce repetitiveness. The top 15 molecular function and biological process categories are represented in Figure 2, and full gene ontology tables can be found in Supplementary Tables S4–S6. Using the Bioconductor package GeneOverlap (v1.32.0), male and female gene lists were compared using a Fisher’s exact test to determine whether there was significant gene overlap of the two lists in comparison to the total number of genes in the mm10 genome.

### 2.5 O4+ separation

The O4 antigen is commonly used to identify oligodendrocyte lineage cells (Sommer and Schachner, 1981), and exists on both OPCs and mature OLGs (Kuhn et al., 2019). Platelet derived growth factor receptor alpha (PDGFRA) and A2B5 are also expressed in OPCs, but antibodies tested for these markers were unable to pull down a sufficient number of cells for downstream analysis (data not shown). Thus, the O4+ marker was chosen to pull down committed oligodendrocyte lineage cells. Frontal pole tissue was used to increase the number of O4+ cells retrieved. Mice were sacrificed by cervical dislocation and decapitation, and the frontal pole was dissected and weighed. Tissue was cut into small pieces and incubated with Accutase (ThermoFisher) at 37°C with 5% CO_2_ for 30 min with gentle agitation every 5 min. Cells were manually dissociated in warmed 10% FBS/HBSS with graded flamed glass pipets and homogenate was passed over a 100 μm filter then centrifuged at 4°C for 10 min at 300×*g*. Cell pellets were resuspended and myelin was removed using myelin removal beads (Miltenyi Biotec), following the manufacturer’s protocol. Magnetic labeling with O4+ beads (Miltenyi Biotec) was used to isolate O4+ cells according to the manufacturer’s protocol. A portion of isolated, myelin removed cells from each sample prior to magnetic bead separation was reserved as an input reference. Input tissue and O4+ pellets were frozen on dry ice and stored at −80°C until use.

### 2.6 RNA isolation

Total RNA from PFC was isolated for qRT-PCR using STAT 60 Reagent (Tel-Test) and RNeasy mini kit (Qiagen) according to manufacturer’s protocol. RNA concentration was determined by absorbance at 260 nm.

### 2.7 Quantitative real-time PCR

Primers for structural components of myelin, transcription factors associated with oligodendrocyte differentiation, and histone methylation regulators with specificity for H3K9me3 were designed and tested for efficiency. Primer sequences can be found in Supplementary Table S2. PFC (Experiment 2), O4+ cells and input (Experiment 3) RNA was reverse transcribed into cDNA using the iScript cDNA kit (Bio-Rad). Real-time PCR was performed using the CFX system (Bio-Rad) for SYBR Green-based detection using manufacturer’s protocol.

The following myelin-related genes were chosen due to their role in myelin formation (Knapp, 1996; Boggs, 2006), myelin integrity (Schafer et al., 2016), or oligodendrocyte processes (Schaeren-Wiemers et al., 2004): myelin basic protein (*Mbp*), proteolipid protein (*Plp*), myelin associated oligodendrocyte basic protein (*Mobp*), myelin-associated glycoprotein (*Mag*), and myelin oligodendrocyte glycoprotein (*Mog*). OLG transcription factors (*Pdgfra*, *Olig1* and *Sox10*) were chosen as markers for the progression of OPC to OLG maturation (Lu et al., 2002; Pozniak et al., 2010; Zhu et al., 2014; Dai et al., 2015a). The epigenetic regulators of H3K9me3, the euchromatic histone lysine methyltransferases, *Ehmt1* and *Ehmt2*, and the lysine histone demethylases, *Kdm4c* and *Kdm4d*, were selected to measure changes in H3K9me3 regulation (Zoabi et al., 2014; Liu et al., 2015; Pack et al., 2016). Biological replicate samples were run in triplicate with cycle thresholds normalized to housekeeping genes *Ppp2r2a* and *Ublcp1* (Wolstenholme et al., 2017). Relative changes in gene expression were normalized to a control male using the delta–delta Ct method (Livak and Schmittgen, 2001). Three-way ANOVAs were used to evaluate the effects of ethanol treatment and timepoint on genes related to myelin structure and oligodendrocyte differentiation with treatment, sex, and time as factors. For Experiment 3, three-way ANOVAs were performed separately for O4 + cells/input and input. Tukey’s posthoc tests were used for *p* < 0.05.

## 3 Results

### 3.1 Adolescent binge ethanol induces differential trimethylation of H3K9me3 at ion channel-related genes in males

Differential trimethylation of H3K9 due to adolescent binge ethanol was assessed with DiffBind. Using a cutoff of *p*-value <0.05 to generate a gene list with a length sufficient for downstream analysis, 691 total genes were differentially bound to H3K9me3 in male PFC (Supplementary Table S3). 402 of these genes were unique to males (Figure 2A) and served as the input for gene ontology analysis. The first 15 molecular function and biological process categories were selected to represent our ChIP-seq gene ontology results. Molecular function categories of genes that were over-represented due to adolescent binge ethanol in males included monoatomic ion channel activity, potassium channel activity, and glycolipid transport. Top biological process categories included circadian behavior, regulation of synapse maturation, and hormone secretion (Figure 2B; Supplementary Table S4).

### 3.2 Adolescent binge ethanol induces differential trimethylation of H3K9 at neuropeptide receptor activity and synapse assembly genes in females

In females, DiffBind analysis of H3K9me3 showed that adolescent binge ethanol caused 1,266 total genes to be differentially bound in female PFC (Supplementary Table S3). 977 genes were uniquely affected in females, and these were used as input for gene ontology analysis (Figure 2A). Molecular function categories that were over-represented due to binge ethanol treatment included MAP kinase phosphatase activity, deacetylase activity, and glutamate receptor activity. Over-represented biological process categories included synapse assembly, presynapse organization, and central nervous system neuron differentiation (Figure 2C; Supplementary Table S5).

### 3.3 Adolescent binge ethanol induces differential trimethylation of H3K9 at synaptic-related genes in both males and females

Of the 691 genes that showed altered H3K9me3 in males and 1,266 genes that showed altered H3K9me3 in females, 289 of those genes were shared between both sexes (Figure 2A). Gene ontology analysis of these 289 genes indicated that they played a role in synaptic transmission and cell signaling. Molecular function categories included glutamate receptor activity, ephrin receptor activity, and transmembrane receptor protein kinase activity. Only 8 biological process categories were identified in our shared set of genes. These biological process categories included presynapse assembly, synaptic membrane adhesion, response to alcohol, and chemical synaptic transmission (postsynaptic) (Figure 2D; Supplementary Table S6). This gene-level functional analysis shows that adolescent binge ethanol produces a striking response of H3K9me3 dysregulation at synapse-related genes.

### 3.4 Adolescent binge ethanol decreases myelin-related gene expression 24 h after the last dose of ethanol

**Figure 3:**
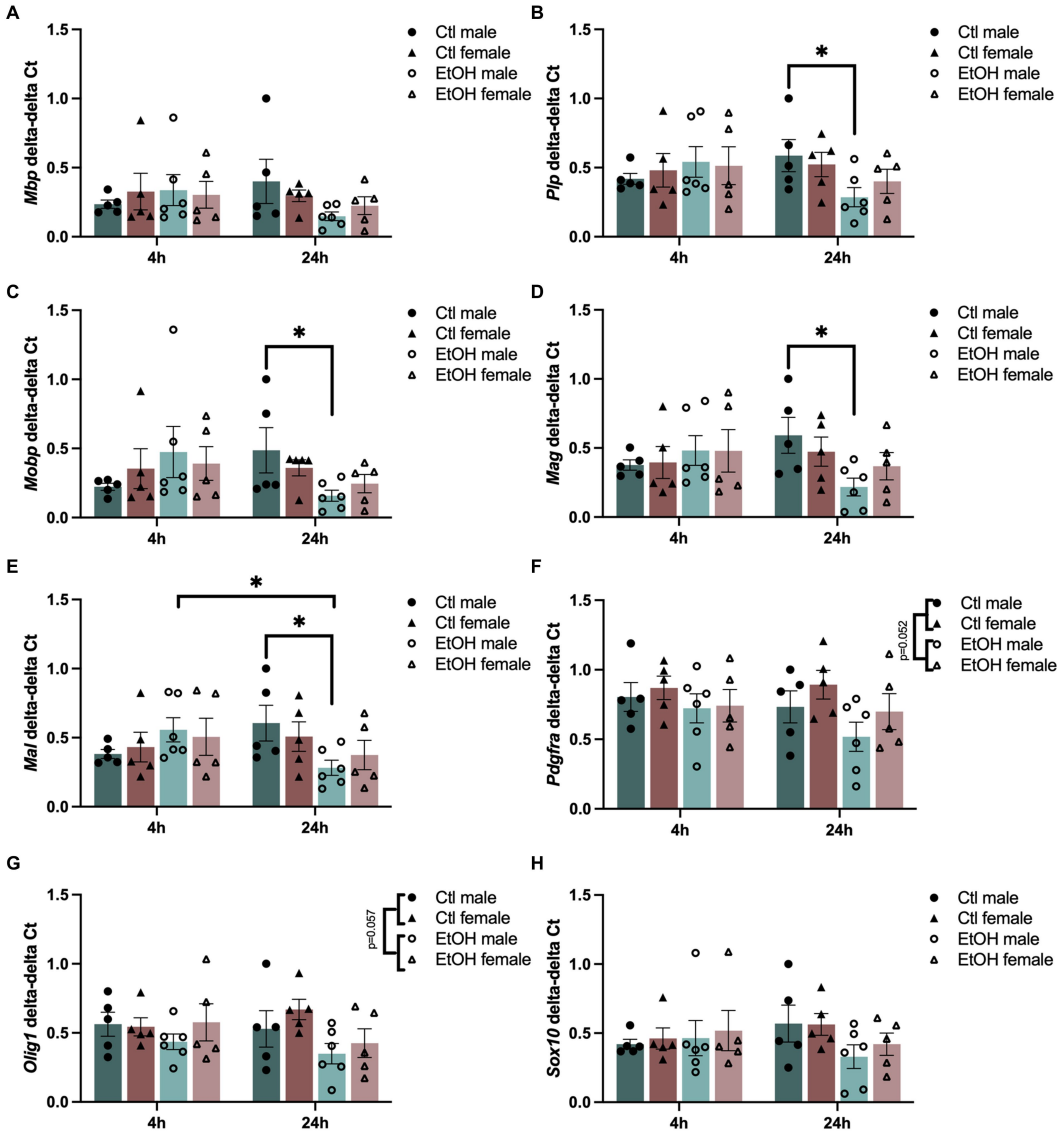
Adolescent binge ethanol decreases myelin and oligodendrocyte-related gene expression in PFC. mRNA expression of **(A)** *Mbp*, **(B)** *Plp*, **(C)** *Mobp*, **(D)** *Mag*, **(E)** *Mal,* **(F)** *Pdgfra,* **(G)** *Olig1,* and **(H)** *Sox10. Plp*, *Mobp*, *Mag*, and *Mal* showed significant interactions between treatment and timepoint by Three-Way ANOVA. *Pdgfra* and *Olig1* showed a trend for a main effect of treatment. ^∗^*p* < 0.05.

We next wanted to determine the expression of genes involved in the transcriptional regulation of oligodendrocyte development (*Pdgfra*, *Olig1, and Sox10*) and the structural components of myelin (*Mbp*, *Plp1*, *Mobp*, *Mag*, and *Mal*) immediately (4 h) and 24 h after the last dose of ethanol. Effects of ethanol treatment and timepoint on genes related to myelin structure and oligodendrocyte differentiation were evaluated using a Three-Way ANOVA with treatment, sex, and time as factors (Figure 3). A significant time by treatment interaction was found for many of the structural components of myelin: *Plp* (F(1, 34)=4.134, *p* = 0.0499), *Mobp* (F(1, 34)=4.754, *p* = 0.0363), *Mag* (F(1, 34)=4.979, *p* = 0.0324) and *Mal* (F(1, 34)=6.424, *p* = 0.0160). Posthoc tests revealed significant interactions between the male control mice versus male ethanol mice at 24 h. Expression of *Plp*, *Mag*, and *Mal* appeared to be decreased by ethanol at 24 h in male mice, but did not survive Tukey’s posthoc. In oligodendrocyte developmental transcription factors, we also noted a trend for a main effect of ethanol treatment in *Pdgfra* (F(1, 34)=4.049, *p* = 0.0522) and *Olig1* (F(1, 34)=3.883, *p* = 0.0570) where ethanol decreased expression. Full ANOVA tables are found in Supplementary Table S7.

### 3.5 Age dependent changes were detected in O4+ enriched cells

**Figure 4:**
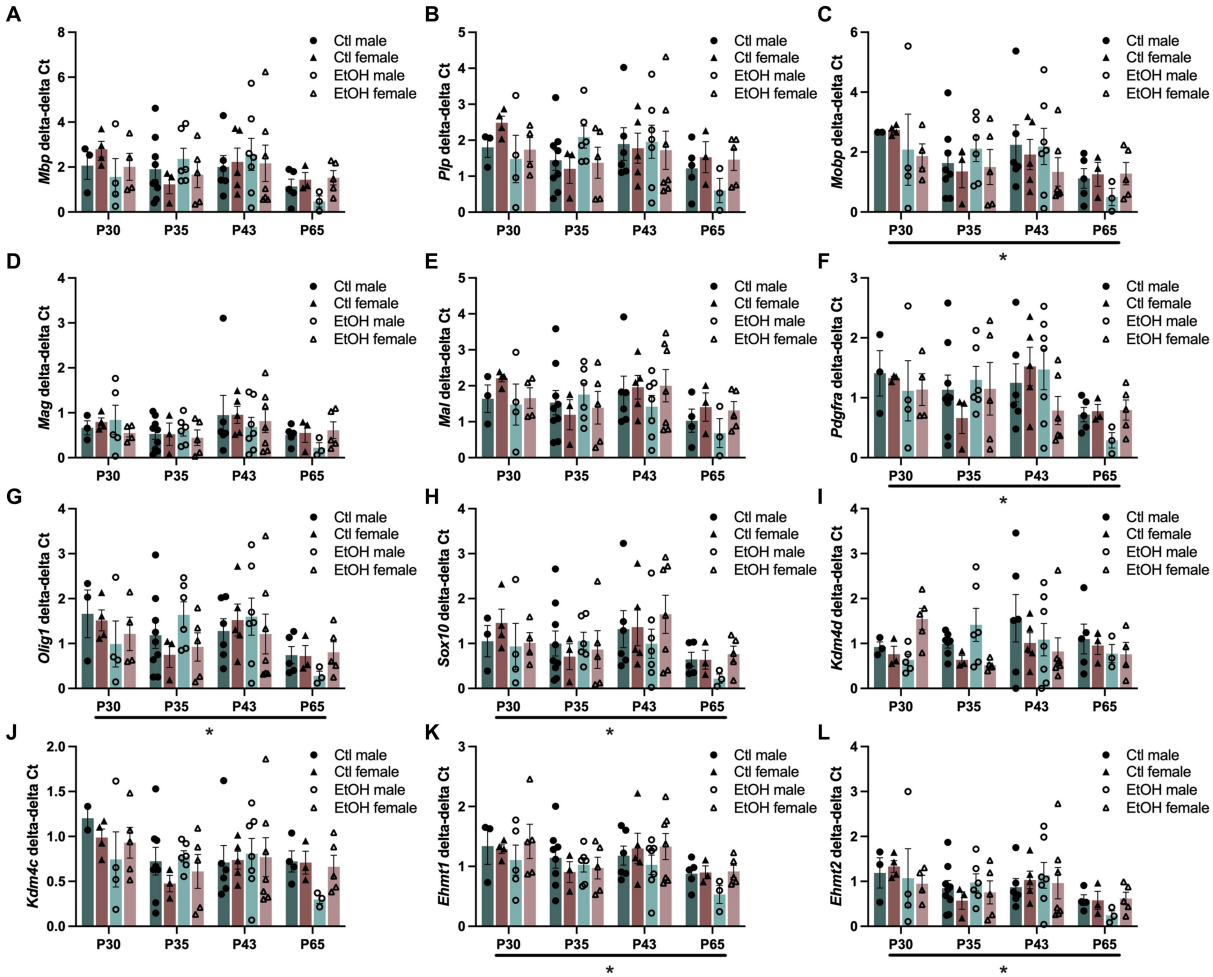
Adolescent binge ethanol does not alter expression of myelin structural genes or transcriptional regulators of OLGs. mRNA expression of **(A)** *Mbp*, **(B)** *Plp*, **(C)** *Mobp*, **(D)** *Mag*, **(E)** *Mal,* **(F)** *Pdgfra,* **(G)** *Olig1,* and **(H)** *Sox10* **(I)** *Kdm4d*, **(J)** *Kdm4c*, **(K)** *Ehmt1* and **(L)** *Ehmt2* in O4+ enriched cells during a time course and ethanol dose response. *Mobp*, *Pdgfra*, *Olig1*, *Sox10*, *Ehmt1* and *Ehmt2* were significantly altered by age. *Mbp*, *Mag*, *Mal*, and *Kdm4c* showed nonsignificant trends to be altered by age. ^∗^*p* < 0.05.

Since ethanol exposure during adolescence decreased expression of transcription factors that play a role in the maturation of OPCs into mature myelin forming OLGs and the expression of genes encoding for the structural components of myelin were reduced in the PFC of males, this experiment was designed to assess how ethanol exposure during adolescence specifically interacts with the progression of oligodendrocyte maturation. To evaluate this, a combined dose response and time course was conducted in OLG cell populations. Tissue was collected from adolescent males and females 24 h after 1, 4, or 8 doses of ethanol (4 g/kg, i.g.), or 3 weeks after mice received all 8 ethanol doses. Cells were magnetically separated from frontal pole tissue into O4+ enriched cell populations and input and qPCR was performed on the same myelin structural components and OLG transcription factors as well as genes specifically involved in the regulation of H3K9me3. Since age is expected to decrease expression of myelin structural components and OLG developmental transcription factors, we analyzed our qPCR as O4+ enriched cells divided by the input. Three-way ANOVAs were performed with treatment, sex, and age as factors and separately for input and O4 + cells/input. The major factor that altered gene expression was age (Figure 4). Three way ANOVAs revealed main effects of age on *Mobp* (*F*(3, 60) = 2.862, *p* = 0.0442), *Pdgfra* (*F*(3, 61) = 2.942, *p* = 0.0400), *Olig1* (*F*(3, 63) = 3.130, *p* = 0.0318), *Sox10* (F(3, 63) = 3.197, *p* = 0.0293), *Ehmt1* (*F*(3, 64) = 4.015, *p* = 0.0111) and *Ehmt2* (*F*(3, 62) = 3.207, *p* = 0.0291) expression where gene expression decreased with age. These effects did not survive Tukey’s posthoc corrections for multiple comparisons. *Mbp* (F(3, 63) = 2.240, *p* = 0.0923), *Mag* (F(3, 64) = 2.263, *p* = 0.0896), *Mal* (*F*(3, 62) = 2.199, *p* = 0.0971), and *Kdm4c* (F(3, 61) = 2.544, *p* = 0.0644) all showed nonsignificant trends to decrease with age. No effects of ethanol treatments, sex or interactions between treatment, sex, and age were found. Full ANOVA results can be found in Supplementary Table S7.

Since our PFC qPCR analysis in Experiment 2 and our previous study (Wolstenholme et al., 2017) have found that expression of many of these myelin structural component genes are decreased by ethanol, we also assessed expression changes of the same genes in the input tissue prior to O4+ enrichment (Supplementary Figure S3). Again, a significant main effect of age was found for most of the genes assessed, where expression generally decreased: *Plp* (*F*(3, 63) = 2.978, *p* = 0.0381), *Mal* (*F*(3, 62) = 3.695, *p* = 0.0163), *Pdgfra* (F(3, 63) = 5.773, *p* = 0.0015), *Olig1* (F(3, 63) = 4.797, *p* = 0.0045), *Sox10* (F(3, 63) = 5.059, *p* = 0.0033), *Kdm4c* (F(3, 63) = 3.854, *p* = 0.0135), *Ehmt1* (F(3, 63) = 3.545, *p* = 0.0194), and *Ehmt2* (F(3, 62) = 3.984, *p* = 0.0116). A trend for a main effect of age was also found for *Mbp* (F(3, 63) = 2.375, *p* = 0.0785). A significant interaction between treatment, age and sex was found for *Ehmt2* expression in input tissue where at PND 35, ethanol females had lower expression than control females and ethanol males (but this did not survive Tukey’s posthoc). Full ANOVA results can be found in Supplementary Table S7.

## 4 Discussion

These studies were designed to interrogate the effects of adolescent binge ethanol on the epigenetic regulation of genes in the frontal cortex with the aim of understanding the dysregulation of oligodendrocyte maturation that may lead to myelin and white matter deficits. ChIP-Seq analysis of H3K9me3 in bulk PFC tissue of both sexes identified differential H3K9me3 at genes involved in synaptic transmission and synaptic function. Males also showed differential H3K9me3 at genes involved in ion channels, potassium channels and synapse maturation. Females, however, showed ethanol-induced H3K9me3 binding at genes involved in synapse assembly, neuron differentiation, and deacetylase activity. Our time course to assess myelin-related gene expression at 4 and 24 h after the last ethanol dose replicated our earlier findings that ethanol decreases myelin-related genes largely 24 h after the last dose and this effect appears to be stronger in males.

Interestingly, we saw that ethanol dysregulated the trimethylation of both H3K9 and H3K36 at about 8% of the same loci in males (176 genes), and about 15% of the same gene loci in females (308 genes). This was surprising given the vastly different functions of the H3K36me3 mark, typically associated with active transcription, and the H3K9me3 mark, typically associated with heterochromatin (Barral et al., 2022). It could be possible that these loci showed changes in both histone methylation marks, as they are transitioning from an active to a repressive state or vice versa due to adolescent binge ethanol treatment. Gene ontology analysis was performed on the genes that overlapped between the H3K36 and H3K9me3 ChIP-seq analyses. In the 176 genes that showed differential trimethylation of both H3K36 and H3K9 in males, top gene ontology categories included transmitter-gated channel activity, channel regulator activity, and synapse assembly. In females, 308 genes showed differential trimethylation of both H3K36 and H3K9 due to adolescent binge ethanol; top gene ontology categories included GPI-linked ephrin receptor activity, glutamate receptor activity, and synapse assembly. Of these 176 genes in males and 308 genes in females that overlapped between the ChIP-seq analyses, 56 of those genes showed differential trimethylation of H3K36 and H3K9 in both males and females. These lists of genes can be found in Supplementary Table S8. Again, gene ontology analysis showed that these genes largely had synaptic-related functions. Adolescent ethanol induced changes in H3K9me3 at neurexin1 (*Nrxn1*) in both males and females, a gene crucial for synaptic assembly (Gomez et al., 2021). This gene was also cryptically transcribed by H3K36me3 in the same tissue (Brocato and Wolstenholme, 2023). One study found that accumulation of H3K9me3 allowed for alternative splicing of *Nrxn1*, and reducing H3K9me3 at *Nrxn1* altered the spliced isoform in the hippocampus, impacting the stability of established memories (Ding et al., 2017). Interestingly, our previous study using the same tissue found that *Nrxn1* also showed differential exon use after adolescent binge ethanol, indicating alternative splicing may be occurring at *Nrxn1* in the PFC following ethanol exposure (Brocato and Wolstenholme, 2023). Dysregulation of *Nrxn1* by adolescent binge ethanol may negatively impact adolescent neurodevelopment in the PFC.

We overlaid our H3K9me3 ChIP data with RNA-seq data that was generated from the same tissue (Brocato and Wolstenholme, 2023) to ask whether there was a pattern between H3K9me3 epigenetic regulation at differentially bound loci and significant differential gene expression due to adolescent ethanol exposure. Given H3K9me3’s repressive role (Kim and Kim, 2012), we expected to find that genes with decreased H3K9me3 due to ethanol would be the same genes where we saw increased gene expression. However, the overlap of our male differentially bound genes (*n* = 691) and male DEGs (*n* = 342 genes at *p* < 0.01) only resulted in 7 genes (*Plcl1, Gnpda2, Tnc, Luzp2, Insig1, Magohb, Dab2*), and the overlap of our female differentially bound genes (*n* = 1,266) and female DEGs (*n* = 376 genes at *p* < 0.01) only resulted in 15 genes (*Tceal6, Ush2a, Ano5, Oprk1, Arhgef6, Cfh, Insig1, Itgb1, Eln, C4b, Car8, Sema3d, Adamts6, Igsf1, Gpr101*). These overlaps show there is little correlation between H3K9me3 differentially bound regions and gene expression. In a survey to map the location of H3K9me3 in human CD4+ cells, Rosenfeld et al. (2009), found that H3K9me3 was not enriched at either genes or in heterochromatin. The presence of H3K9me3 at the 3′ end of a gene (as opposed to the gene body or promoter) was associated with decreased expression, but not strongly, suggesting that H3K9me3 may play a role in chromatin organization (Rosenfeld et al., 2009).

We also predicted that ethanol would decrease H3K9me3 at more genes rather than induce H3K9me3, given how ethanol interacts with the methionine metabolism pathway to influence methylation reactions (Brocato and Wolstenholme, 2021). Ethanol is known to cause a decrease in methylation through its inhibition of enzymes responsible for producing S-adenosylmethionine, the sole methyl donor for all methylation reactions in the cell (Brocato and Wolstenholme, 2021). However, we found that in both males and females, a majority of ethanol-induced changes to H3K9me3 led to increased binding of genes in the ethanol exposed mice. These unexpected results may be due to ethanol’s ability to alter H3K9me3-mediated chromatin regulation and gene expression through separate processes that are not necessarily linked. Ethanol can impact chromatin regulation through its metabolites (Mews et al., 2019; Brocato and Wolstenholme, 2021), impacts a number of receptor systems (Abrahao et al., 2017), and can also move intracellularly to impact a number of intracellular proteins (Abrahao et al., 2017). While changes in chromatin accessibility are typically associated with corresponding changes to gene expression, ethanol may not produce such predictable changes given its impact on multiple pathways and cell processes. For example, ethanol may reduce methylation by reducing the pool of S-adenosylmethionine present for methylation reactions to occur, which would reduce levels of H3K9me3. Simultaneously, ethanol may impact certain transcription factors, such as CREB (Yang et al., 1998; Soares-Simi et al., 2013; Zhang et al., 2018) to reduce gene expression levels at specific genes. In this case, even if H3K9me3 is reduced, transcription factors or other important cell signaling molecules could be negatively impacted by ethanol and unable to initiate transcription, even when chromatin is left accessible by the lack of H3K9me3. Further studies are needed to uncover this complicated relationship of chromatin regulation and gene expression in the presence of ethanol.

Initially, we hypothesized that adolescent binge ethanol would induce H3K9me3 occupancy at specific membrane excitability-related genes, given the data presented in Liu et al. (2015). Liu et al. showed that H3K9me3 must be present at specific genes regulating membrane excitability to reduce their gene expression and allow oligodendrocyte precursor cells to mature into myelin-forming oligodendrocytes (Liu et al., 2015). While we did not see H3K9me3 changes in these specific genes (Supplementary Table S3), this could be due to the tissue type used in the Liu et al. (2015) study—while we used adolescent mouse prefrontal cortex tissue, H3K9me3 changes were found by Liu et al. (2015) at membrane excitability related genes in cultured OPCs or OLGs taken from rat cortex (PND 1). Furthermore, it is possible that changes in H3K9me3 at genes directly related to oligodendrocyte differentiation were not detectable in bulk tissue. Performing single-cell ChIP-seq or ChIP-seq in O4+ cells would allow us to gain a better understanding of the genes that H3K9me3 regulates in OPCs and OLGs, and provide evidence that ethanol acts through H3K9me3 to reduce myelin-related gene expression. It is also possible that H3K9me3 may be regulating other membrane excitability related genes in adolescent mice. In our ChIP-seq study, we found that adolescent ethanol altered trimethylation of H3K9 at other ion channels, glutamate receptors, and synaptic proteins in both males and females, including *Grin3a, Gria4, Grid2, Grik2, Grm3, Glra3, Kcnh7, Nrxn1,* and *Cntn5* (Supplementary Table S3). The gene expression values for these genes from our previous RNA-seq study (Brocato and Wolstenholme, 2023) are shown in Supplementary Figure S4. Specifically, *Kcnh7*, *Nrxn1* and *Cntn5* have been associated with myelin formation or phenotypes (Boscia et al., 2021; Dauar et al., 2022; Sebastian et al., 2022), and glutamate signaling has been shown to be important to oligodendrocyte development and survival (Kougioumtzidou et al., 2017; Turan et al., 2021). Changes to the regulation of these genes and others involved in synaptic function could impact the progression of oligodendrocyte maturation and play a role in ethanol-induced myelin reduction.

We noted some consistent differences in ethanol’s transcriptional response between the sexes. Our ChIP-Seq data identified some overlaps in genomic regions differentially bound by H3K9me3 in both sexes, but a majority of the genes were differentially bound in one sex or the other. In the qPCR time course studies, we found that many of the myelin structural component genes were mostly altered in the males. Similarly, at PND 65 in our O4+ enriched cells, males with a history of binge ethanol decreased expression of OLG transcriptional regulators and myelin structural components. These sex differences could be due to the developmental differences between males and females—male brains have been shown to mature at a slower rate than females (Drzewiecki et al., 2017). As our ethanol administration is based on postnatal day, and females are further along in brain development at the onset of our dosing paradigm, ethanol may not impact females OLG development as strongly as it affects males.

We replicated our earlier findings in regard to decreased myelin expression (Wolstenholme et al., 2017), showing that adolescent binge ethanol treatment significantly decreased *Plp*, *Mobp*, *Mag*, and *Mal* in males twenty four hours after the last binge. We have also extended these findings, potentially showing ethanol’s early impact on transcription factors necessary for oligodendrocyte development. The transcription factors, *Pdgfra* and *Olig1*, showed a trend towards an ethanol-induced decrease in gene expression which was stronger at 24-h (Figure 3). As *Olig1* regulates transcription of major myelin-specific genes including *Mag* and *Plp* (Li and Richardson, 2008), and *Pdgfra* has been shown to be a major determinant of oligodendrocyte differentiation (Zhu et al., 2014), this ethanol-induced early decrease in gene expression could lead to the later significant decrease in gene expression of *Plp*, *Mobp*, *Mag*, and *Mal* at the 24-h mark. These early reductions in *Olig1* and *Pdgfra* could be indicative of a delay in OLG maturation in ethanol exposed mice. As a result, genes involved in later development of myelin structure (i.e., *Plp*, *Mag*, and *Mal*) could be less abundant due to fewer mature myelin forming OLGs.

In our study to directly investigate the progression of oligodendrocyte development using O4+ enriched cell populations from the mouse frontal pole, we measured gene expression of the same myelin structural components (*Mbp*, *Plp*, *Mobp*, *Mag*, and *Mal*) and transcription factors (*Pdgfra*, *Olig1* and *Sox10*), as well as genes that regulate H3K9me3 (*Kdm4c*, *Kdm4d*, *Ehmt1* and *Ehmt2*). We expected to find a dose dependent alteration in these genes during our binge ethanol paradigm. However, we only detected a main effect of age where, in general, a decrease in gene expression for most of these genes was found at later ages. As expected, this maturational decline was also reflected in the input tissue used for the O4+ enrichment studies. We did not detect any changes in gene expression due to increasing ethanol binges, even when the data was normalized for the maturational decline. At PND 65, three weeks following the last ethanol binge, we do note a general trend for ethanol to decrease expression of these genes in male O4+ cells as compared to control males suggesting a lasting effect of ethanol’s transcriptional dysregulation of OLG maturation in O4+ cells in the frontal cortex. This change in OLG transcriptional regulators and myelin structural components was not seen in the input tissue, nor in females (see Figure 4).

There are a few possible reasons we may not have been able to detect robust changes in gene expression in our O4+ enriched cell studies. First, we collected these cells from mouse frontal pole which includes large white matter tracts such as the corpus callosum and anterior commissure as well as grey matter structures (the prefrontal cortex, nucleus accumbens caudate putamen, etc.). These regions have highly heterogeneous population of cells and amounts of white matter and myelin, and all have different OLG maturation rates. Liu et al. (2021) showed that qPCR in bulk tissue was not able to detect changes in myelin related gene expression, even though brain regional effects of myelin proteins were found in adults following adolescent intermittent ethanol (Liu et al., 2021). Second, there is a possibility that the magnetic antibody sorting and tissue processing altered cellular processes such as transcription. This could be a likely possibility, since gene expression changes from the input tissue did not replicate our prior ethanol findings from bulk PFC tissue snap frozen immediately after harvest (in Experiment 2 and Wolstenholme et al., 2017). Third, the choice of O4+ as a marker to collect late OPC progenitor cells to mature myelin-forming OLGs may either not be as selective as desired, or may not enrich for all of these cell types. This possibility is less likely since we do see enrichment of these myelin-related genes as compared to input tissue. Overall, we found that while binge ethanol in adolescence decreased gene expression of myelin related genes in male PFC tissue, our O4+ enrichment studies were unable to detect such changes when using larger brain regions and studies are needed at more discrete brain regional resolution. The trend for a lasting decrease in the transcriptional regulation and expression of myelin structural genes coupled with our H3K9me3 differential binding at genes that regulate synaptic activity suggest that binge ethanol during adolescence can lead to lasting changes in the regulation, development and maturation of OLGs leading to decreased white matter in adulthood. These studies have identified potential target genes and signaling pathways for further study as potential therapeutic strategies for intervention.

## 5 Conclusion

Overall, this study unveiled potential genes that may be contributing to adolescent binge ethanol-related myelin loss. As H3K9me3 plays an important role in oligodendrocyte differentiation (Liu et al., 2015), and H3K9-related gene expression was previously found to be decreased in the PFC after adolescent binge ethanol (Wolstenholme et al., 2017), we assessed ethanol-induced changes to this histone mark in the developing adolescent PFC. We further assessed ethanol-induced changes at the transcription level with qPCR time course approaches in O4+ cells to assess oligodendrocyte progenitor and oligodendrocytes specifically. Through this set of experiments, we found that adolescent binge ethanol altered H3K9me3 regulation of synaptic-related genes, and genes specific for glutamate and potassium channels. Given the data presented in Liu et al. (2015), dysregulation of ion channels could impede oligodendrocyte differentiation required for ongoing myelin development in the PFC throughout adolescence. Our gene expression findings support our previous work; that adolescent binge ethanol decreased expression of myelin-related genes in the PFC of male animals, 24 h after the last dose of ethanol. We further expanded this finding by looking at an earlier time point, potentially showing ethanol’s early impact on transcription factors necessary for oligodendrocyte development. This early change in gene expression in transcription factors associated with oligodendrocyte differentiation may lead to the later significant decrease in myelin-related gene expression at *Mag*, *Mal*, and *Plp* at the 24-h mark. The O4+ enrichment time course and dose response studies were inconclusive, but could suggest lasting dysregulation of OL maturation at the transcriptional level. Further experiments will be required to assess the exact role of these genes in ethanol-induced myelin loss, and whether they are regulated by histone marks other than H3K9me3.

## Data availability statement

The original contributions presented in the study are publicly available. The datasets generated and analyzed for this study have been deposited with the Gene Expression Omnibus resource (GSE220746 with data subsets ChIP-seq: Accession #GSE220742 and RNA-seq: Accession #GSE220745).

## Ethics statement

The animal study was approved by Virginia Commonwealth University Institutional Animal Care and Use Committee. The study was conducted in accordance with the local legislation and institutional requirements.

## Author contributions

EB: Conceptualization, Formal analysis, Investigation, Methodology, Validation, Visualization, Writing – original draft, Writing – review & editing. RE: Investigation, Methodology, Writing – review & editing. AM: Investigation, Visualization, Writing – review & editing. MK: Investigation, Methodology, Validation, Writing – review & editing. GL: Formal analysis, Investigation, Writing – review & editing. JW: Conceptualization, Formal analysis, Investigation, Methodology, Project administration, Resources, Supervision, Validation, Visualization, Writing – review & editing.

## Supplementary material

SUPPLEMENTARY FIGURE S1 H3K9me3 ChIP antibody validation and sequencing coverage. **(A)** H3K9me3 antibody (Abcam) was validated using positive (*Serp1a*) and negative (*Rpl30*) primers to visualize regions of expected high and low H3K9me3, respectively, and was compared to IgG (Millipore) to identify enrichment over non-specific binding. Graph shows representative control male sample. **(B)** Representative H3K9me3-ChIP read coverage of three genes that showed differential trimethylation of H3K9: *Grid2*, *Grik2*, and *Nrxn1*.

SUPPLEMENTARY FIGURE S2 Peak annotation of genes differentially bound by H3K9me3 in males **(A)** and females **(B)** due to ethanol. Percentages are rounded to the nearest tenth.

SUPPLEMENTARY FIGURE S3 qPCR time course and dose response in input tissue. mRNA expression of **(A)** *Mbp*, **(B)** *Plp*, **(C)** *Mobp*, **(D)** *Mag*, **(E)** *Mal,* **(F)** *Pdgfra,* **(G)** *Olig1,* and **(H)** *Sox10* **(I)** *Kdm4d*, **(J)** *Kdm4c*, **(K)** *Ehmt1* and **(L)** *Ehmt2* in input cells during a time course and ethanol dose response. *Plp*, *Mal*, *Pdgfra*, *Olig1*, *Sox10*, *Kdm4c*, *Ehmt1*, and *Ehmt2* were significantly altered by age. A trend for a main effect of age was also found for *Mbp*. A significant interaction between treatment, age and sex was found for *Ehmt2* expression at PND 35; ethanol females had significantly lower expression than control females and ethanol males. ∗ p < 0.05.

SUPPLEMENTARY FIGURE S4 Relative expression of synaptic-related genes differentially bound by H3K9me3 due to ethanol. Log2 fold change expression values were determined using RNA-seq analysis of the same PFC tissue (Brocato and Wolstenholme, 2023) in males **(A)** and females **(B)**.
