# Supplementary figures and images for: Adolescent binge ethanol impacts H3K9me3-occupancy at synaptic genes and the regulation of oligodendrocyte development
Source: Front Mol Neurosci. 2024 May 22;17:1389100. doi: 10.3389/fnmol.2024.1389100 (PMC11150558; doi:10.3389/fnmol.2024.1389100)

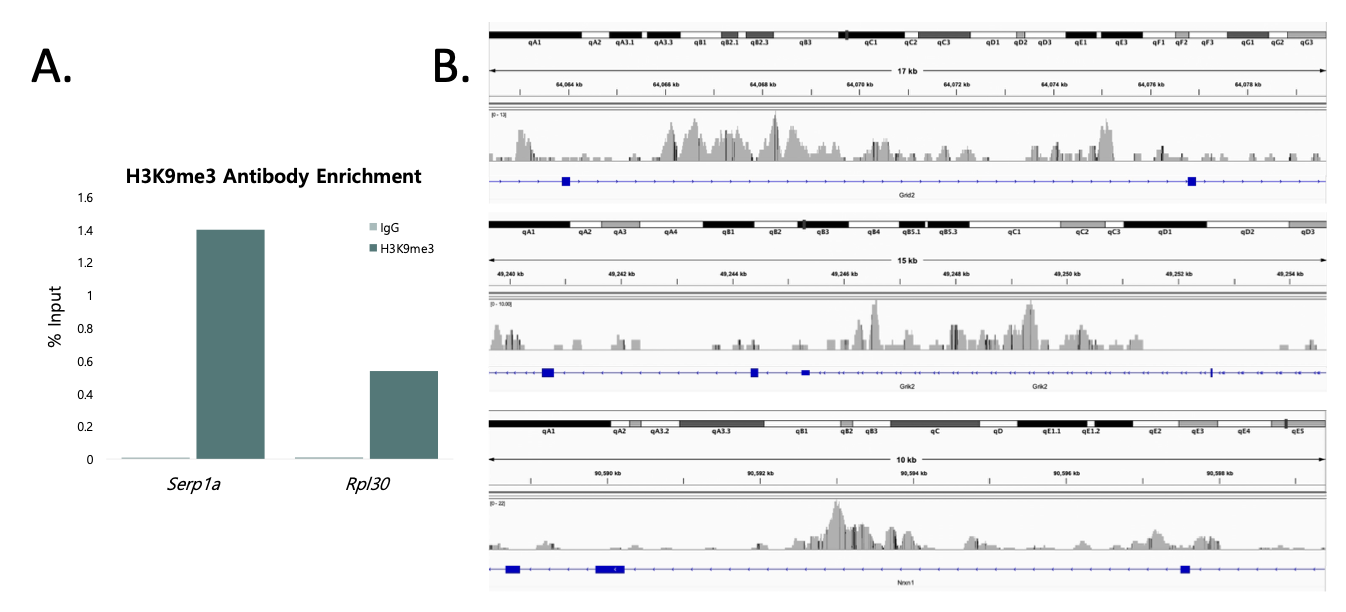

Supplement: SUPPLEMENTARY FIGURE S1 — H3K9me3 ChIP antibody validation and sequencing coverage. (A) H3K9me3 antibody (Abcam) was validated using positive (Serp1a) and negative (Rpl30) primers to visualize regions of expected high and low H3K9me3, respectively, and was compared to IgG (Millipore) to identify enrichment over non-specific binding. Graph shows representative control male sample. (B) Representative H3K9me3-ChIP read coverage of three genes that showed differential trimethylation of H3K9: Grid2, Grik2, and Nrxn1. [file Image_1.PNG]

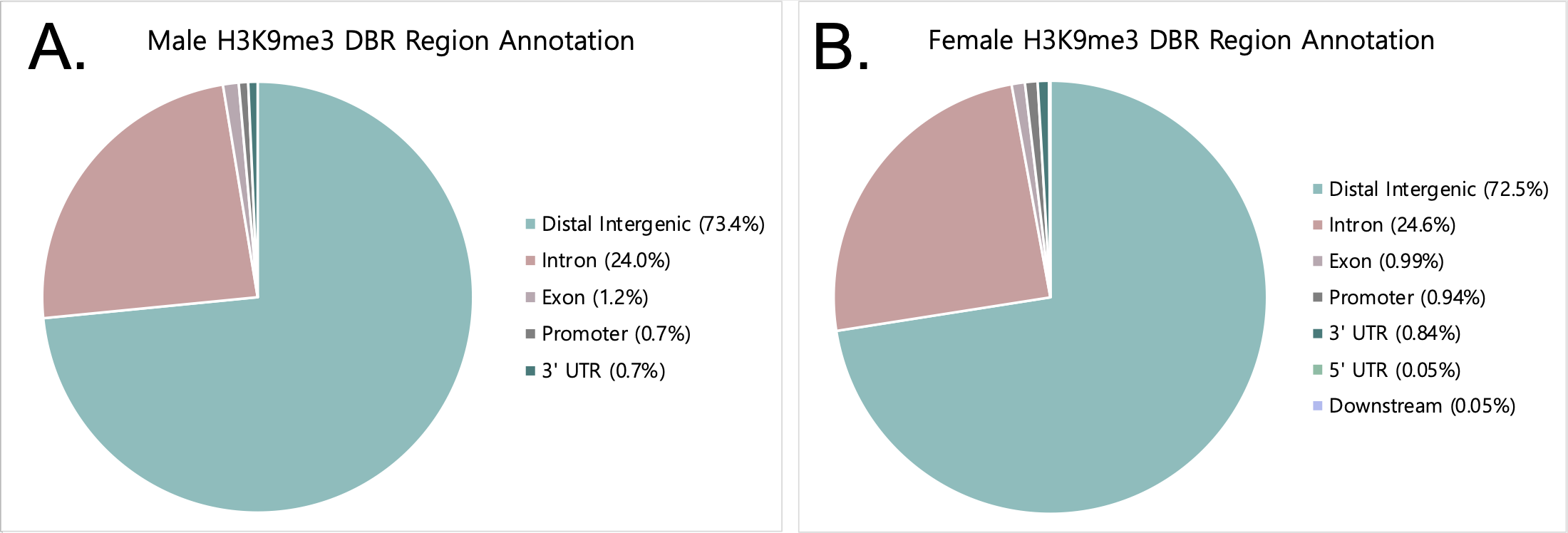

Supplement: SUPPLEMENTARY FIGURE S2 — Peak annotation of genes differentially bound by H3K9me3 in males (A) and females (B) due to ethanol. Percentages are rounded to the nearest tenth. [file Image_2.TIF]

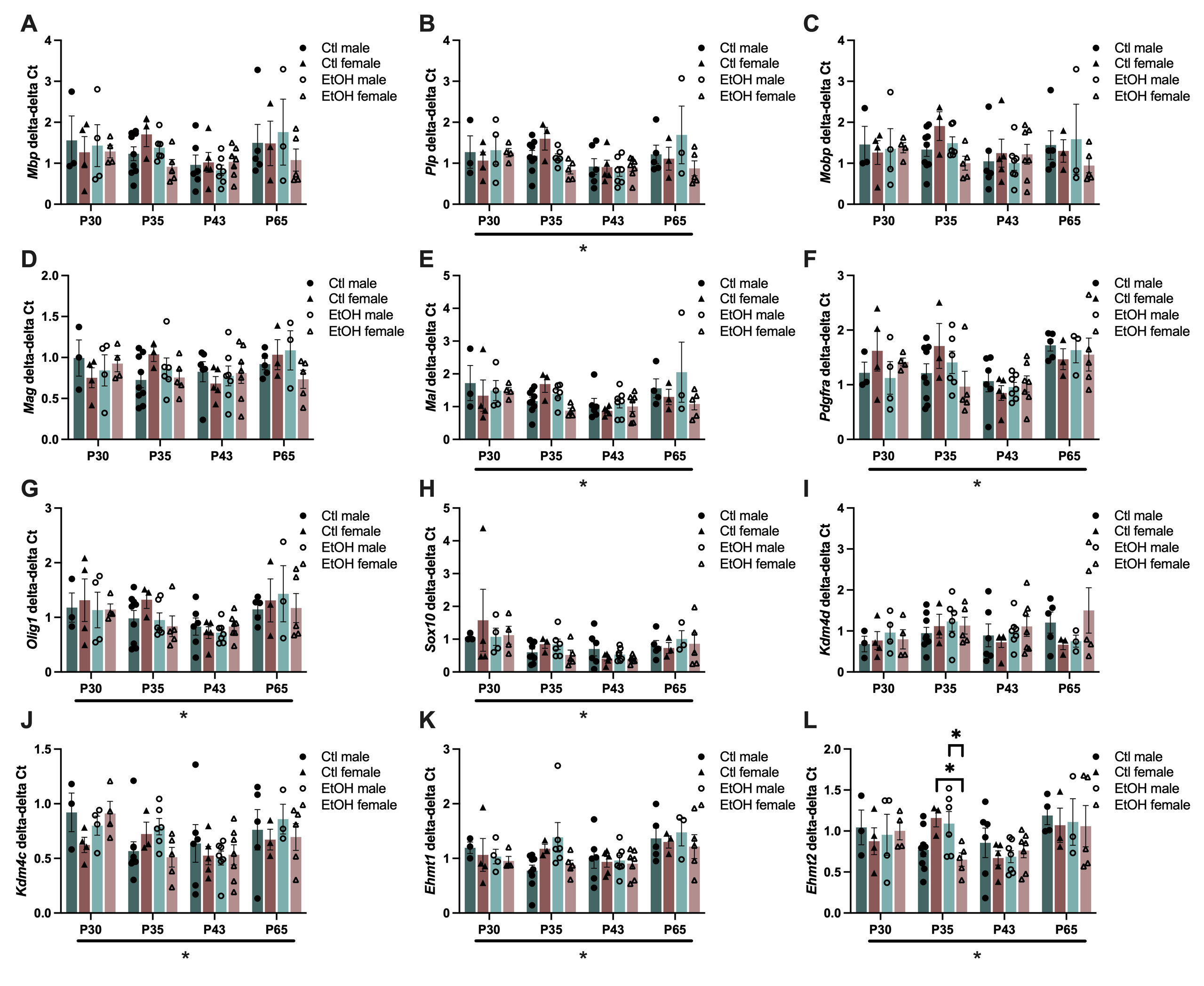

Supplement: SUPPLEMENTARY FIGURE S3 — qPCR time course and dose response in input tissue. mRNA expression of (A) Mbp, (B) Plp, (C) Mobp, (D) Mag, (E) Mal, (F) Pdgfra, (G) Olig1, and (H) Sox10 (I) Kdm4d, (J) Kdm4c, (K) Ehmt1 and (L) Ehmt2 in input cells during a time course and ethanol dose response. Plp, Mal, Pdgfra, Olig1, Sox10, Kdm4c, Ehmt1, and Ehmt2 were significantly altered by age. A trend for a main effect of age was also found for Mbp. A significant interaction between treatment, age and sex was found for Ehmt2 expression at PND 35; ethanol females had significantly lower expression than control females and ethanol males. ∗ p < 0.05. [file Image_3.TIF]

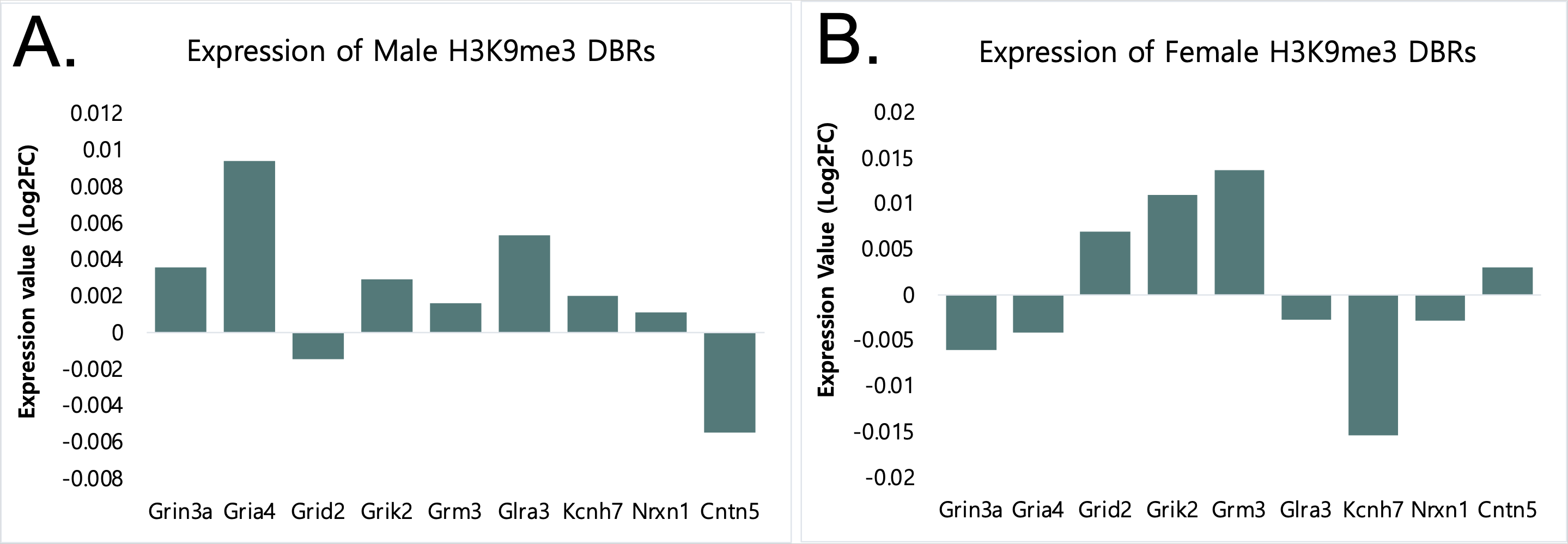

Supplement: SUPPLEMENTARY FIGURE S4 — Relative expression of synaptic-related genes differentially bound by H3K9me3 due to ethanol. Log2 fold change expression values were determined using RNA-seq analysis of the same PFC tissue (Brocato and Wolstenholme, 2023) in males (A) and females (B). [file Image_4.TIF]
